# Supplementary material for: Different Forms of Disorder in NMDA-Sensitive Glutamate Receptor Cytoplasmic Domains Are Associated with Differences in Condensate Formation
Source: Biomolecules. 2022 Dec 20;13(1):4. doi: 10.3390/biom13010004 (PMC9855357; doi:10.3390/biom13010004)

**Figure S1.**Confirmation of the protein components within LLPS droplets.

To confirm the protein composition within the condensed droplets formed during LLPS, we fluorescently labeled individual components and performed multicolor imaging. PSD-95 was labeled with Alexa 488; the CTD2 domain from GluN2B (CTD2B) was labeled with Alexa 647 while CC-PBM from synGAP was unlabeled. No droplet formation occurs without CC-PBM. The ternary mixture contained a 1:1:1 ratio of PSD-95, CTD2B and CC-PBM at 20 µM, which included 300 nM of each labeled protein. Shown are representative images of the ternary mixture from Differential Interference Contrast (DIC) microscopy (left); from excitation at 488 nm to detect PSD-95 (center) and excitation at 642 nm excitation to detect CTD2B (right). This shows that all droplets detected by DIC contain both PSD-95 and CTD2, which agrees well with sedimentation analysis (Figure 4B).


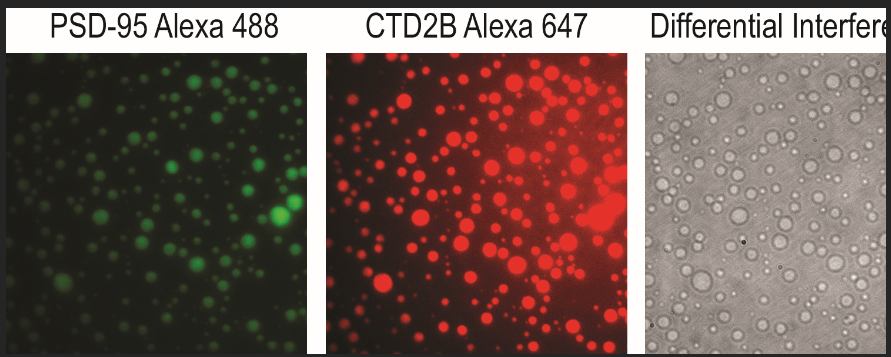

Supplement: Supplementary file 1 [file biomolecules-13-00004-s001.zip › biomolecules-2014925-Supplementary Figure S1.docx]
